# Mechanisms of miR-3189-3p-mediated inhibition of c-MYC translation in triple negative breast cancer

**DOI:** 10.1186/s12935-022-02620-z

**Published:** 2022-05-31

**Authors:** Cecilia Vittori, Duane Jeansonne, Hassan Yousefi, Celeste Faia, Zhen Lin, Krzysztof Reiss, Francesca Peruzzi

**Affiliations:** 1 Louisiana State University Health Sciences Center and Stanley S. Scott Cancer Center, 1700 Tulane Ave, New Orleans, LA USA; 2 Department of Biochemistry, Louisiana State University Health Sciences Center, 533 Bolivar St., New Orleans, LA USA; 3 Department of Pathology and Laboratory Medicine, Tulane University Health Sciences Center and Tulane Cancer Center, 1700 Tulane Ave, New Orleans, LA USA

**Keywords:** 4EBP1, miR-3189-3p, Translation, c-MYC, Breast cancer

## Abstract

**Background:**

Triple negative breast cancer (TNBC) is an aggressive subtype of breast cancer characterized by the lack of estrogen receptor, progesterone receptor, and HER2. Our lab previously characterized miR-3189-3p as a microRNA with potent anti-cancer activity against glioblastoma. Here, we hypothesized a similar activity in TNBC cells. As miR-3189-3p is predicted to target a variety of RNA binding proteins, we further hypothesized an inhibitory effect of this miRNA on protein synthesis.

**Methods:**

MDA-MB-231 and MDA-MB-468 cells were used to investigate the effect of miR-3189-3p on cell proliferation, migration, and invasion. TGCA database was used to analyze the expression of miR-3189-3p, c-MYC, 4EPB1, and eIF4E in breast cancer. Western blotting and RT-qPCR assays were used to assess the expression of selected proteins and RNAs after transfections.

**Results:**

Although c-MYC is not a predicted gene target for miR-3189-3p, we discovered that c-MYC protein is downregulated in miRNA-treated TNBC cells. We found that the downregulation of c-MYC by miR-3189-3p occurs in both normal growth conditions and in the absence of serum. The mechanism involved the direct inhibition of eIF4EBP1 by miR-3189-3p. Additionally, we found that miR-3189-3p could negatively affect cap-independent translation mediated by internal ribosome entry sites (IRES) or by m6A. Finally, miR-3189-3p sensitized TNBC cells to doxorubicin.

**Conclusion:**

Overall, results indicated that miR-3189-3p exerts its anti-tumor activity through targeting translational regulatory proteins leading to an impairment in c-MYC translation, and possibly other oncogenic factors, suggesting that miR-3189-3p, alone or in combination, could be a valuable therapeutic approach against a malignancy with few treatment options.

## Introduction

Breast cancer is the second leading cause of cancer death in women [1, 2]. Although mortality has been declining, there are still about 1.3 million cases being diagnosed annually worldwide. Triple negative breast cancer (TNBC) is characterized by the lack of estrogen receptor, progesterone receptor, and HER2 and cannot be treated with the available hormone therapies and receptor targeted treatments. Currently, even if some TNBCs do benefit from immunotherapy and PARP inhibitors [3, 4], surgery and chemotherapy appear to remain the first-line treatments. Therefore, there is a clear need to identify novel therapeutic targets for the treatment of TNBC.

The eukaryotic initiation factor 4E (eIF4E)-binding protein 1 (eIF4EBP1, 4EBP1) belongs to the family of cap-dependent translation repressor proteins. 4EBP1 blocks cap-dependent translation through the inhibitory binding of eIF4E, a protein essential for the initiation of translation [5–7]. Phosphorylation of 4EBP1 by kinases such as mTORC1, CDK1, and PIM kinases inhibits binding to eIF4E, thereby allowing the initiation of cap-dependent translation [7, 8]. Although this protein is a cap-dependent translation repressor and therefore should be tumor suppressive, 4EBP1 is upregulated in numerous cancers, including TNBC [9–12]. However, the molecular details underlying the potential oncogenic activity of 4EBP1 are still unclear.

The oncogene c-MYC, a regulatory gene involved in cell growth, metabolism, differentiation, and apoptosis, is often increased in TNBCs, making it an attractive therapeutic target [13–15]. MicroRNAs (miRNAs) are small, non-coding RNA molecules that regulate gene expression. Although miRNAs have been identified as key players in cancer pathogenesis, the specific miRNA-mediated pathways involved in TNBC are still largely unknown. MiR-3189 was identified by next generation sequencing (NGS) performed on paired normal and tumor breast tissue [16], although its expression in either normal or tumor tissue was not reported [16]. Previously, our lab found that miR-3189-3p mimic exerts anti-tumoral effects via inhibition of migration and proliferation of glioblastoma cells [17]. Although c-MYC is not a predicted gene target for miR-3189-3p, in this study we found a strong downregulation of c-MYC in TNBC cells following transfection with miR-3189-3p. This novel mechanism involves the miRNA-mediated inhibition of 4EBP1 expression, resulting in downregulation of the cap-dependent and cap-independent translation of c-MYC under normal growth conditions, as well as under stress conditions. This is important, since current anticancer therapies often fail to be effective in the tumor microenvironment where cells adapted to survive in stress conditions, such as hypoxia and low nutrients. Altogether, our findings further validate miR-3189-3p as a potential therapeutic that regulates c-MYC in TNBC, a malignancy for which there are few treatment options.

## Materials and methods

### Cell culture, transfection, and reagents

MDA-MB-231, MDA-MB-468, and hTERT-HME1 (HME1) cells were obtained from the American Type Culture Collection (ATCC, Manassas, VA) and cultured under standard growth conditions (DMEM supplemented with 10% FBS). For plasmid or plasmid/miRNA co-transfection experiments, cells were seeded at a density of 4 to 6 × 10^5^ cells/60 mm dish, and transfected using Lipofectamine 2000 (Invitrogen, Waltham, MA) per manufacturer’s instructions. The miR-3189-3p mirVana miRNA mimic was purchased from Ambion (Thermo Fisher Scientific, Waltham, MA), and used at a final concentration of 50 nM. When only the miRNA was transfected, we used RNAiMAX (Invitrogen, Waltham, MA). For the experiments in serum-free medium (SFM, DMEM supplemented with 0.1% BSA), 72 h post-transfection cells were washed twice with Hank’s balanced salt solution (HBSS) followed by the addition of SFM for 24 h. 4EBP1 and eIF4E siRNAs and controls were purchased from Santa Cruz Biotechnology (Santa Cruz, CA), and were used at the final concentration of 50 nM. The proteasome inhibitor MG-132 was from Calbiochem (Millipore Sigma, Burlington, Massachusetts) and was used at the final concentration of 1 μM. The vector expressing HPV16 circular RNA-encoded E7 protein was obtained by cloning HPV16 E7 circular RNA fragment into the pcDNA3.1 (+) circRNA mini vector [18] and the empty control vector (Addgene) were a gift of Dr. Zhen Lin. For this experiment, HPV16 circular RNA vector or empty vector were transfected together with the miR-3189-3p mimic with the transfection conditions described above.

### Cell proliferation assay

MDA-MB-231 cells were plated at a density of 8.0 × 10^4^ cells/well in a 96-well plate overnight and transfected with non-targeting control or miR-3189-3p mimic. Cells were harvested and counted by trypan blue exclusion at T0 (day of transfection), 24, 48, 72, and 96 h post-transfection (n = 4/each time point). Proliferation of MDA-MB-468 was performed as described above plating 9.0 × 10^4^ cells/well, while HME1 cells were plated at 4.0 × 10^4^ cells/well. For co-transfection experiments, MDA-MB-231 cells were transfected first with miR-3189-3p using RNAiMAX. 16 h later cells were transfected with either pcDNA3 or pcDNA3/4EBP1 plasmids using Lipofectamine 2000. Cells were harvested 24 h following plasmid transfection, counted and seeded in quadruplicate at the concentration of 6,000/well in a 96 well plate. Cells were counted by trypan blue exclusion at 24, 48, 72, and 96 h after plating.

### Scratch assay

MDA-MB-231 cells were plated in a 35 mm glass bottom dish (MatTek Corporation, Ashland, MA) at a density of 1.8 × 10^5^ cells/dish and allowed to adhere overnight. Following transfection with miR-3189-3p for 72 h, the scratch assay was performed by moving a pipette tip across the cell monolayer. Migration of MDA-MB-231 cells into the cell-free area was monitored for up to 24 h using live cell time-lapse imaging (VivaView FL incubator fluorescent microscope, Olympus, Center Valley, PA).

### Migration and invasion assays

For migration assays, TNBC cells were transfected with miR-3189-3p or scramble control using Lipofectamine RNAiMAX. After 72 h, cells were detached from the plate and seeded into the 6.5 mm transwell insert (8.0 μm pore size; Corning, NY) at the final concentration of 3 × 10^4^ cells per chamber in 200 μl of serum-free medium. 600 μl of complete culture medium supplemented with 10% FBS as chemoattractant were added to the lower chamber and cells were incubated at 37 °C. After 24 h, the inserts were gently washed 3 times with cold PBS and cells were fixed using 100% methanol. Non-migrated cells were removed from the top of the inserts using cotton swabs. After fixation, the cells migrated on the bottom of the insert were washed again with PBS and stained with 0.4% crystal violet. Migrated cells were counted from three random fields per insert at the magnification of 20× and averaged from at least three biological replicates. For the invasion assay we used Corning BioCoat Matrigel Invasion Chambers with 8.0 μm PET Membrane from Corning (Corning, NY). The protocol for invasion was essentially the same as the migration assay, except that we plated 8 × 10^4^ MDA-MB-231 cells or 10 × 10^4^ MDA-MB-468 cells per chamber in a total of 500 μl of serum-free medium, and 750 μl of complete culture medium containing 10% FBS as chemoattractant were added to the lower chamber.

### MTT assays

Cells were transfected with scramble control or miR-3189-3p 48 h before plating them into 96 well plate at the concentration of 8 × 10^3^ cells per well and incubated for 24 h. Then, the cells were treated with 9 different concentrations of doxorubicin (25, 10, 5, 2.5, 1, 0.5, 0.1, 0.05, and 0.01 µM). After incubation for 48 h, 20 μl of 5 mg/ml MTT (Sigma) dissolved in PBS was added to the cells. After 2 h at 37 °C, dimethyl sulfoxide (DMSO) was added and the absorbance at 570 nm was measured using a BioTek SYNFRGY neo2 microplate reader (BioTek, Winooski, VT, USA).

### Western blots

Cells were lysed in modified RIPA lysis buffer (50 mM Tris, pH 7.4, 150 mM NaCl, 1% NP-40, 1 mM EGTA, pH 7.4, 0.25% sodium deoxycholate), supplemented with 1 mM PMSF, 1 mM sodium orthovanadate, and phosphatase and protease inhibitor cocktails (Sigma, St. Louise, MO). Whole-cell lysates (30 to 70 µg) were separated on a 4–15% SDS-PAGE gel (Mini-PROTEAN TGX Precast Gel, Bio-Rad, Hercules, CA) and transferred to a 0.2 μm nitrocellulose (Trans-Blot Turbo Mini 0.2 µm Nitrocellulose Transfer Packs, Bio-Rad, Hercules, CA) using the Trans-Blot TURBO apparatus (Bio-Rad, Hercules, CA). GAPDH and 14-3-3ζ antibodies were from Santa Cruz Biotechnology (Santa Cruz, CA) and were used as loading controls. Anti-c-MYC, 4EBP1, and eIF4E antibodies were purchased from Cell Signaling Technology (Beverly, MA).

### Quantitative RT-PCR

**Fig. 1:**
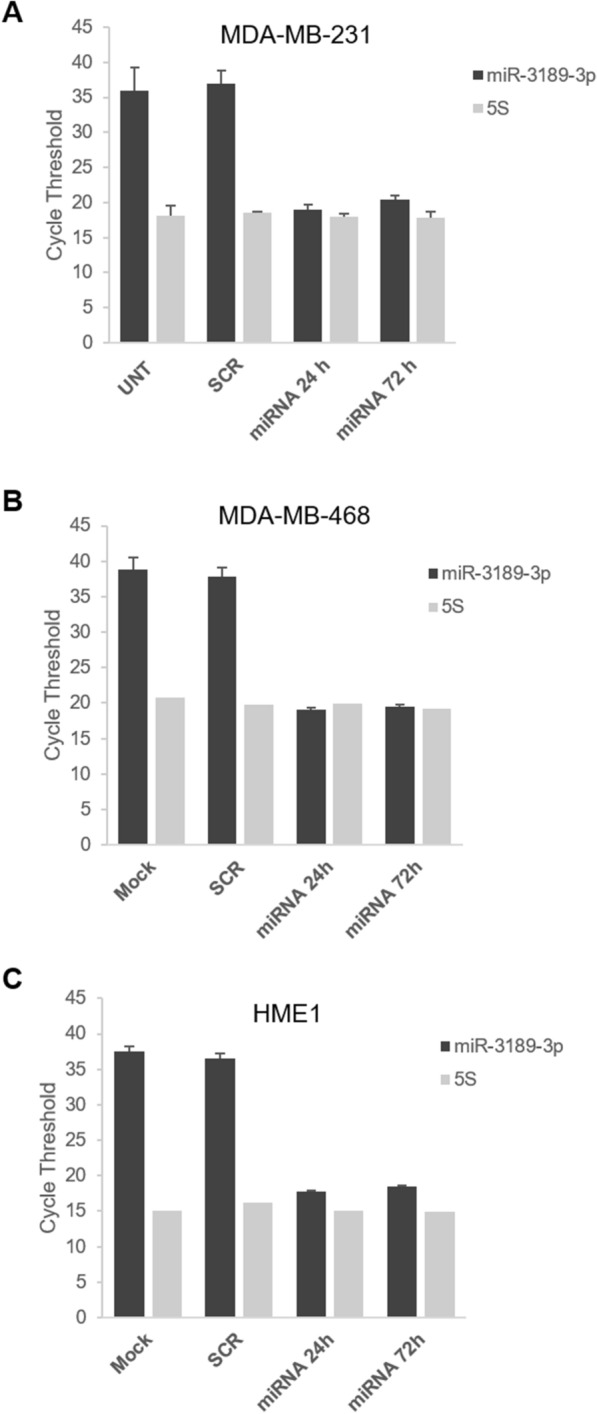
Relative expression of miR-3189-3p in TNBC after transfection with the miRNA mimic. Bar graphs showing cycle threshold values of mature miR-3189-3p expression in MBA-MD-231 (**A**), MDA-MB-468 (**B**), and HME1 (**C**) cells at 24 and 72 h post-transfection. For reference, expression of the small RNA 5S is also indicated

Total RNA was isolated using the mirVana miRNA extraction kit (Ambion, Thermo Fisher Scientific). 500 ng of total RNA were reverse transcribed using the High Capacity cDNA Reverse Transcription kit for mRNAs (Applied Biosystems, Waltham, MA). Quantitative real-time PCR was performed using a Roche LightCycler 480 Real-Time PCR System (Indianapolis, IN). Each sample was assessed in duplicate and GAPDH or 5S small RNA were used as reference genes for mRNA or miRNA, respectively. The relative quantification of gene expression was calculated using the comparative Ct (2^−ΔΔCt^) method, as described in our previous publications (17–19). Note that we could not use this method to determine the relative expression of miR-3189-3p in miRNA-transfected TNBC cells compared to control-transfected cells, since the cycle threshold values (Cts) before transfection were > 35 (Fig. 1).

### Human clinical data and correlation analyses

The RNASeq data relative to Breast Invasive Carcinoma samples were downloaded from the METABRIC dataset on the TCGA data portal (http://www.cbioportal.org/). Gene expression data from 1904 breast tumors (Expression log intensity levels (Illumina Human v3 microarray)) and clinical information data from METABRIC [19, 20] were used as well. According to the PAM50 classification [21], METABRIC breast cancer dataset was divided into 5 subtypes, including the Basal/claudin-low (triple negative breast cancer, n = 401), HER2 + (n = 220), Lum A (n = 679), Lum B (n = 461) and Normal-like (n = 140) subtypes. The expression analyses of all transcripts were performed using the student’s t-test. Pearson's correlation analysis was used for the analysis of the correlation of gene expression.

### Cloning and mutagenesis of the eIF4EBP1 3′UTR

The genomic sequence corresponding to the 3′UTR of eIF4EBP1 was PCR-amplified from MDA-MB-231 cells. This PCR product was inserted into the multiple cloning site downstream of the *Renilla* luciferase reporter gene in the psiCHECK-2 vector (Promega). The primers in the 3′UTR sequence of eIF4EBP1 gene used were: forward, 5′-CCGCTCGAGATGGACATTTAAAGCACCAGCC ATC, containing the XhoI restriction site (underlined) and reverse, 5′-ATAAGAATGCGGCCGC CTTGGCCCTAGGGCGAAGG, containing the NotI restriction site (underlined).

Mutation of the miR-3189-3p putative binding site in the *eIF4EBP1* 3′UTR sequence was generated using the QuikChange Lightning site-directed mutagenesis kit (Agilent Technologies, Santa Clara, CA) using the psiCHECK2/3′UTR plasmid as a template. The oligonucleotides used were: 5′-AGGAGCTGCCACCCCTTCCGGAGTGACCCCTGCC, and the complementary sequence 5′-GGCAGGGGTCACTCCGGAAGGGGTGGCAGCTCCT, which contains the mutated bases in the miR-3189-3p binding site (underlined).

### Dual luciferase assay

MDA-MB-231 cells were plated at a density of 8 × 10^4^ cells/well in a 12-well plate and transfected with psiCHECK-2 vector expressing the *eIF4EBP1* 3′UTR (160 ng/well) alone, the 3′UTR with the miR-3189-3p mimic (50 nM), or the binding site mutant 3′UTR with the miRNA mimic using Lipofectamine RNAiMAX. Cells were harvested 24 h post transfection and protein lysates were assayed for luciferase activity (Dual-Luciferase reporter assay system, Promega). Relative quantification was determined by normalizing Renilla values with the firefly luciferase internal control. Data represent the average of at least three independent experiments, each in duplicate.

### Cloning of c-MYC IRES into pYIC plasmid

The pYIC vector was obtained from Addgene (Watertown, MA). The plasmid contains a bicistronic fluorescent reporter gene in which EYFP expression is driven by a cap-dependent mechanism while ECFP translation is under the control of a 587 bp EMCV-IRES sequence. The 395 bases 5′UTR of human c-MYC (c-MYC mRNA sequence accession number: NM_002467.6) was amplified by PCR and cloned into the pYIC vector by the Klenow approach, after removal of the EMCV-IRES sequence through digestion with EcoRI and BstXI restriction enzymes. The primers used for the cloning were: forward 5′GCCTGACTGACTAAGTAATTCCAGCGAGAGGCAGA-3′ and reverse 5′-TTGCTCACCA TGGTTGTCGCGGGAGGCTGCT. For Western blot analysis, HA-tagged ECFP from EMCV-IRES-ECFP or c-MYC-IRES-ECFP constructs was detected using an anti-HA antibody (Cell Signaling Technology).

### Statistical analysis

Data are presented as mean ± S.D. All data were graphed using GraphPad Prism and Excel software packages. Comparison between two experimental groups was performed using Student’s t test. One way ANOVA was used to compare three or more groups. p ≤ 0.05 were considered statistically significant.

## Results

### MiR-3189-3p impairs growth, migration, and invasion of TNBC

**Fig. 2:**
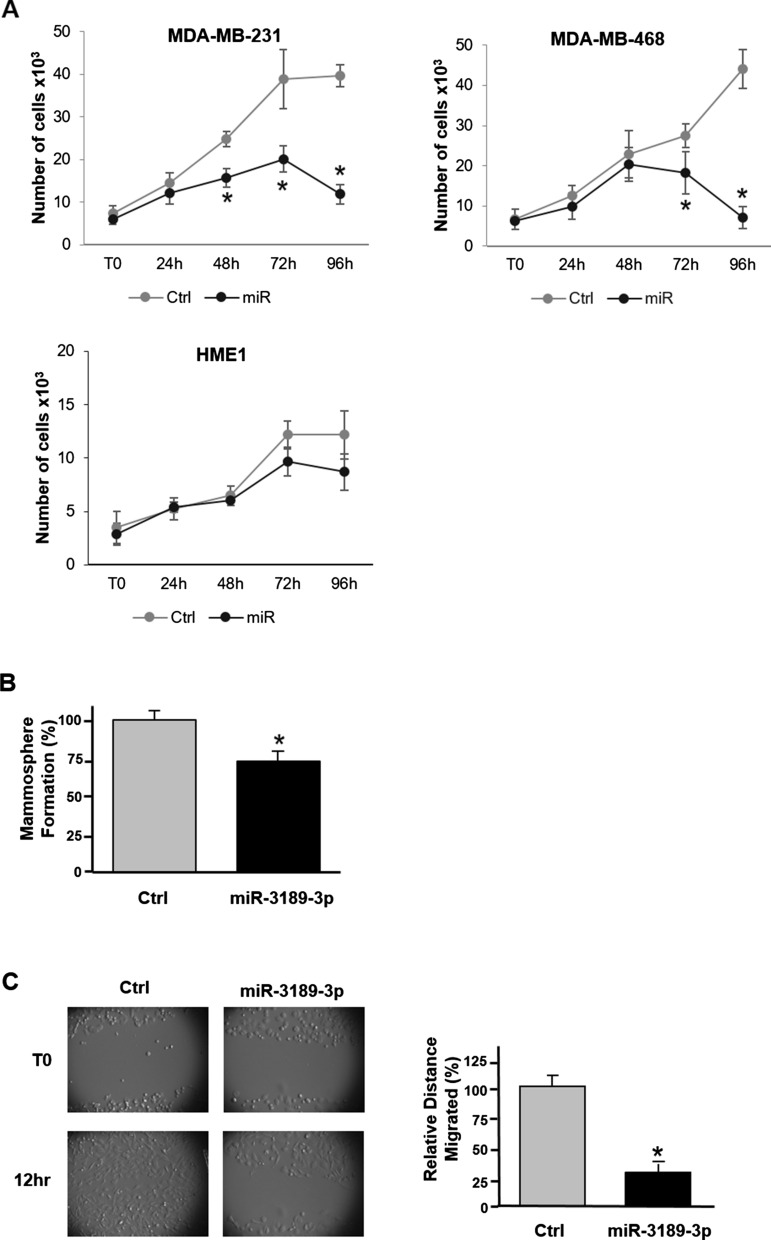
MiR-3189-3p impairs proliferation and migration of TNBC cells. **A** Plot indicating proliferation of MDA-MB-231, MDA-MB-468, and HME1 cells at the indicated time points. **B** Cell proliferation assay for mammospheres obtained from MDA-MB-231 cells, performed 72 h post-transfection with the miR-3189-3p or the scramble control. Results are expressed as percent of the number of mammospheres obtained from cells transfected with miR-3189-3p versus the number of mammospheres from control cells. **C** Representative images of a scratch assay 72 h post-transfection with miR-3189-3p or scramble control. Migration of cells toward the cell-free area was monitored by time-lapse imaging using an Olympus VivaView system. Original magnification 10×. The bar graph represents quantification of the migration obtained from three experiments, each in duplicate. In all graph, the asterisks indicate statistical significance

**Fig. 3:**
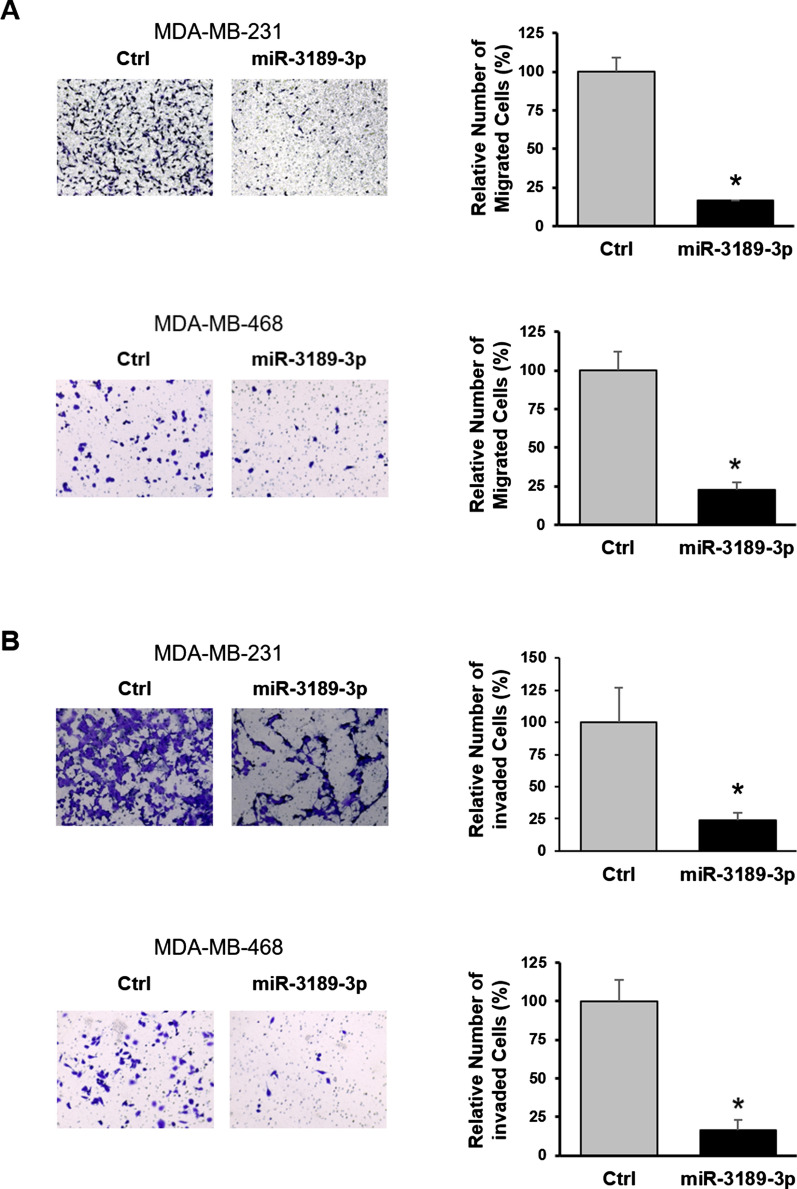
MiR-3189-3p impairs migration/invasion of TNBC cells.** A** Representative images of a transwell migration assay performed using MDA-MB-231 (upper panel) and MDA-MB-468 (lower panel) 72 h post-transfection with the miR-3189-3p or scramble control (Ctrl). Migrated cells were stained with crystal violet and pictures were acquired with an optical microscope. Original magnification 20×. Quantification of the migrated cells was obtained from three different random image fields per insert and averaged from at least three biological replicates. **B** Representative images of a transwell invasion assay performed using MDA-MB-231 (upper panel) and MDA-MB-468 (lower panel) and analyzed 72 h post-transfection with the miR-3189-3p or scramble control (Ctrl). Transwells were stained and pictures were acquired with an optical microscope. Original magnification 20×. The bar graph indicates the average number of cells migrated through the Matrigel calculated from three different random image fields per insert and averaged from at least three biological replicates. * Indicates statistical significance

In our previous study, we demonstrated that miR-3189-3p has a strong anti-cancer activity against glioblastoma [17]. We have now extended these findings to triple-negative breast cancer where miR-3189-3p was found to inhibit proliferation, migration, and invasion of MDA-MB-231 cells. Similar to glioblastoma cells, we did not detect miR-3189-3p in the TNBC cell lines MDA-MB-231 and MDA-MB-468 nor in control normal epithelial cells HME1 (Cycle threshold (Ct) > 35; Fig. 1). However, upon transfection with miR-3189-3p mimic, the Ct values for the miRNA were comparable to those of the 5S small RNA (Fig. 1) at both 24 and 72 h, indicating a robust and stable presence of the miRNA compared to untransfected, mock-, or scramble-transfected cells. To determine the effect on proliferation, MDA-MB-231 cells were transfected with miR-3189-3p mimic and allowed to grow for up to 96 h. Cell proliferation was measured by trypan blue exclusion at T0, 24, 48, 72, and 96 h post-transfection. Results show a statistically significant inhibition in proliferation of about 50% and 80% at 72 and 96 h, respectively, of cells transfected with miR-3189-3p mimic when compared to control cells transfected with a non-targeting control RNA (Fig. 2A, left panel). In the same experimental setting MDA-MB-468 also showed a reduction of about 50 and 70% in proliferation compared to the scramble control (Fig. 2A, right panel). On the other hand, transfection of miR-3189-3p of the normal breast epithelial HME1 cells, did not result in a significant decrease in proliferation when compared to scramble control (Fig. 2A, lower panel). Furthermore, in a 3D model of cancer growth, miR-3189-3p also slowed the proliferation of mammosphere (generated from MDA-MB-231cells) by approximately 25% compared to controls (Fig. 2B). We then evaluated cell migration using an in vitro model of wound healing (scratch assay). While control-transfected cells completely filled the gap within 12 h, we observed 75% decrease in the ability of miR-transfected cells to migrate across the scratched area in the same amount of time (Fig. 2C). These data were further confirmed in a transmigration assay, in which overexpression of miR-3189-3p reduced the ability of MDA-MB-231cells and MDA-MB-468 to migrate across the pores of the filter of about 80% and 75%, respectively, when compared to cells transfected with the scramble control (Fig. 3A).

To further investigate the effect of miR-3189-3p on cell invasion, we transfected TNBC cells with the miRNA or the scramble control and analyzed the ability of the transfected cells to invade Matrigel coated transwells. Results showed that the number of invaded cells in the miR-transfected MDA-MB-231 or MDA-MB-468 was decreased of about 75% and 80%, respectively, when compared to the control transfected cells (Fig. 3B). Altogether, these experiments demonstrate the ability of miR-3189-3p to inhibit migration and invasion of both MDA-MB-231 and MDA-MB-468 cells, suggesting a potential use of this miRNA against TNBC.

### MiR-3189-3p inhibits c-MYC translation by targeting 4EBP1

**Fig. 4:**
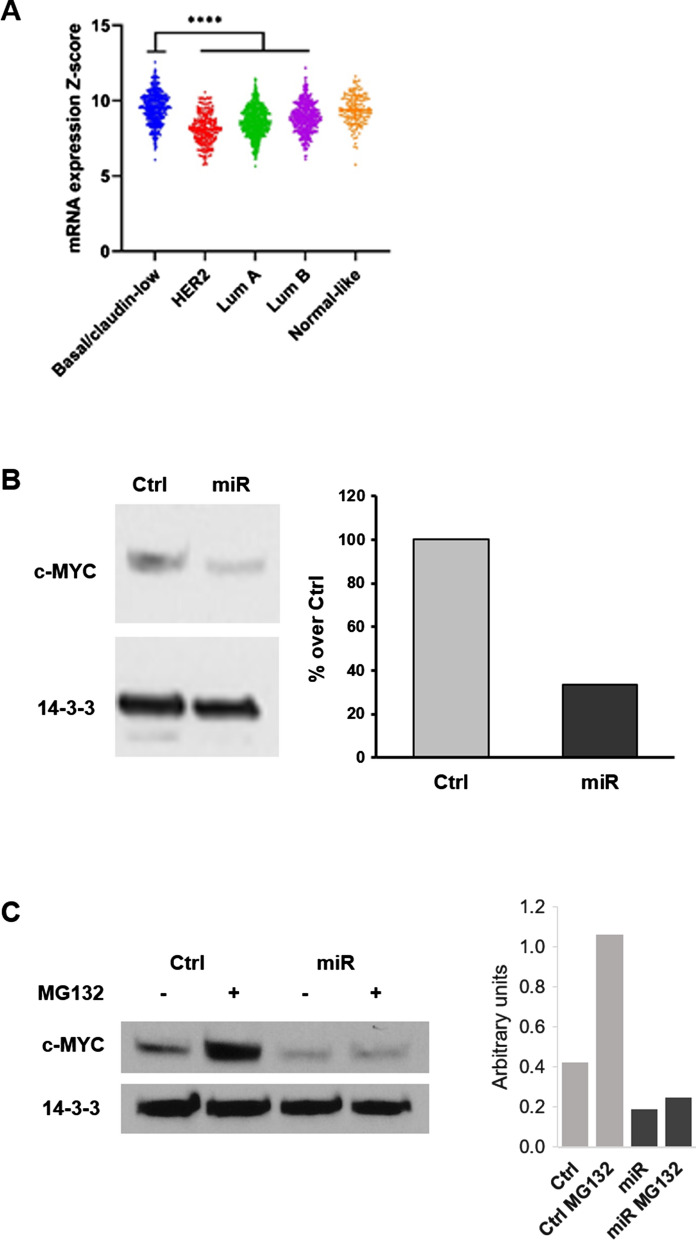
miR-3189-3p mediates c-MYC downregulation at translational level. **A** Expression of c-MYC mRNA in different subtypes of breast cancer (METABRIC). * Indicates statistical significance. **B** Western blot for c-MYC protein from MDA-MB-231 cells transfected with miR-3189-3p (miR) or scramble control (Ctrl) for 72 h. Densitometric analysis of the bands relative to c-MYC was done using Image J. **C** Western blots performed on MDA-MB-231 cells that were transfected with scramble control (Ctrl) or miR-3189-3p (miR) in the absence or presence of the proteasome inhibitor MG132. 14–3-3 was used as loading control

C-MYC is a highly expressed gene that plays an important role in supporting the malignant phenotype of TNBC [13, 15]. Accordingly, our genomic data analysis from the Molecular Taxonomy of Breast Cancer International Consortium METABRIC [19, 20] showed that c-MYC mRNA has the highest expression level in TNBC compared to the other breast cancer subtypes (Fig. 4A), while we could not find expression of miR-3189-3p in any cancer tissue annotated in the TGCA data base. Considering the number of predicted gene targets with a function in RNA metabolism, we decided to investigate whether miR-3189-3p could affect c-MYC expression, even if c-MYC mRNA is not a predicted target for this miRNA. MDA-MB-231 cells were transfected with miR-3189-3p and collected after 72 h for protein and RNA analysis. Western blot analysis showed lower levels of c-MYC protein expression in miR-transfected cells as compared to controls (Fig. 4B). On average, a 70% ± 5% reduction in c-MYC protein is observed in cells overexpressing miR-3189-3p (Fig. 4B, right panel). Interestingly, quantitative PCR (qPCR) analysis from cells transfected with miR-3189-3p revealed no change in MYC mRNA expression (data not shown), suggesting a post-transcriptional regulation of c-MYC by the miRNA.

We decided next to determine whether miR-3189-3p induces a reduction of c-MYC protein levels through the proteasome pathway in order to increase the rate of protein degradation. Cells were transfected with miR-3189-3p or control RNA for 48 h. After this time, cells were incubated in the absence or presence of the proteasome inhibitor MG-132 (1 µM) for an additional 24 h. Figure 4C shows that MG132 treatment prevented c-MYC downregulation in control transfected cells but it was not effective on stabilizing c-MYC in cells expressing miR-3189-3p. Altogether, these findings suggest that miR-3189-3p mediated c-MYC downregulation is independent from the proteasome pathway.

**Fig. 5:**
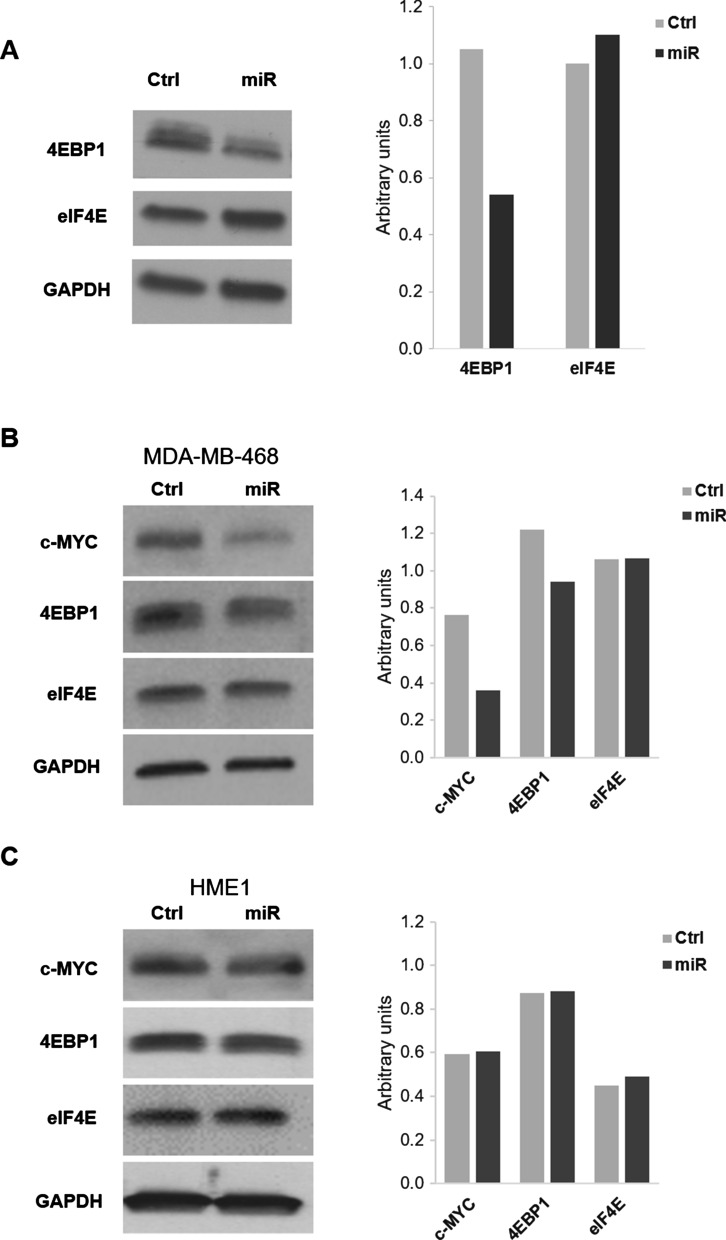
Overexpression of miR-3189-3p downregulates c-MYC and 4EBP1 protein levels in TNBC cells but not in normal epithelial cells. **A** Western blot to detect 4EBP1 and eIF4E proteins in MDA-MB-231 cells transfected with miR-3189-3p (miR) or scramble control (Ctrl). **B**, **C** Representative Western blots showing expression of c-MYC, 4EBP1, and eIF4E in MDA-MB-468 (b) and HME1 normal epithelial cells. GAPDH was used as loading control

Therefore, we turned our attention to miR-3189-3p targets that may mediate c-MYC translation. Analysis of miRNA gene target prediction programs revealed that 4EBP1, musashi RNA-binding protein 2 (MSI2), and eIF4E are potential targets of miR-3189-3p (Targetscan 7.2), although MSI2 and eIF4E prediction rank low in the cumulative weighted context scores [22]. As 4EBP1 and eIF4E have been shown to be involved in the translation of c-MYC [6], they were selected for further studies. Expression of miR-3189-3p in MDA-MB-231 cells resulted in reduced levels of 4EBP1 protein compared to control-transfected cells (Fig. 5A). Expression levels of total MSI2 (data not shown) and eIF4E did not change in the same experimental conditions (Fig. 5A) indicating that these genes are not validated targets of the miRNA, at least in these cells. Next, we evaluated expression levels of c-MYC, 4EBP1, and eIF4E in MDA-MB-468 (Fig. 5B) as well as in the normal epithelial cells HME1 (Fig. 5C) after ectopic expression of miR-3189-3p. Results shown a miRNA-induced downregulation of c-MYC, 4EBP levels in MDA-MB-468 but not in the HME1 cell line, in which no significant changes were observed for the expression of the selected proteins.

**Fig. 6:**
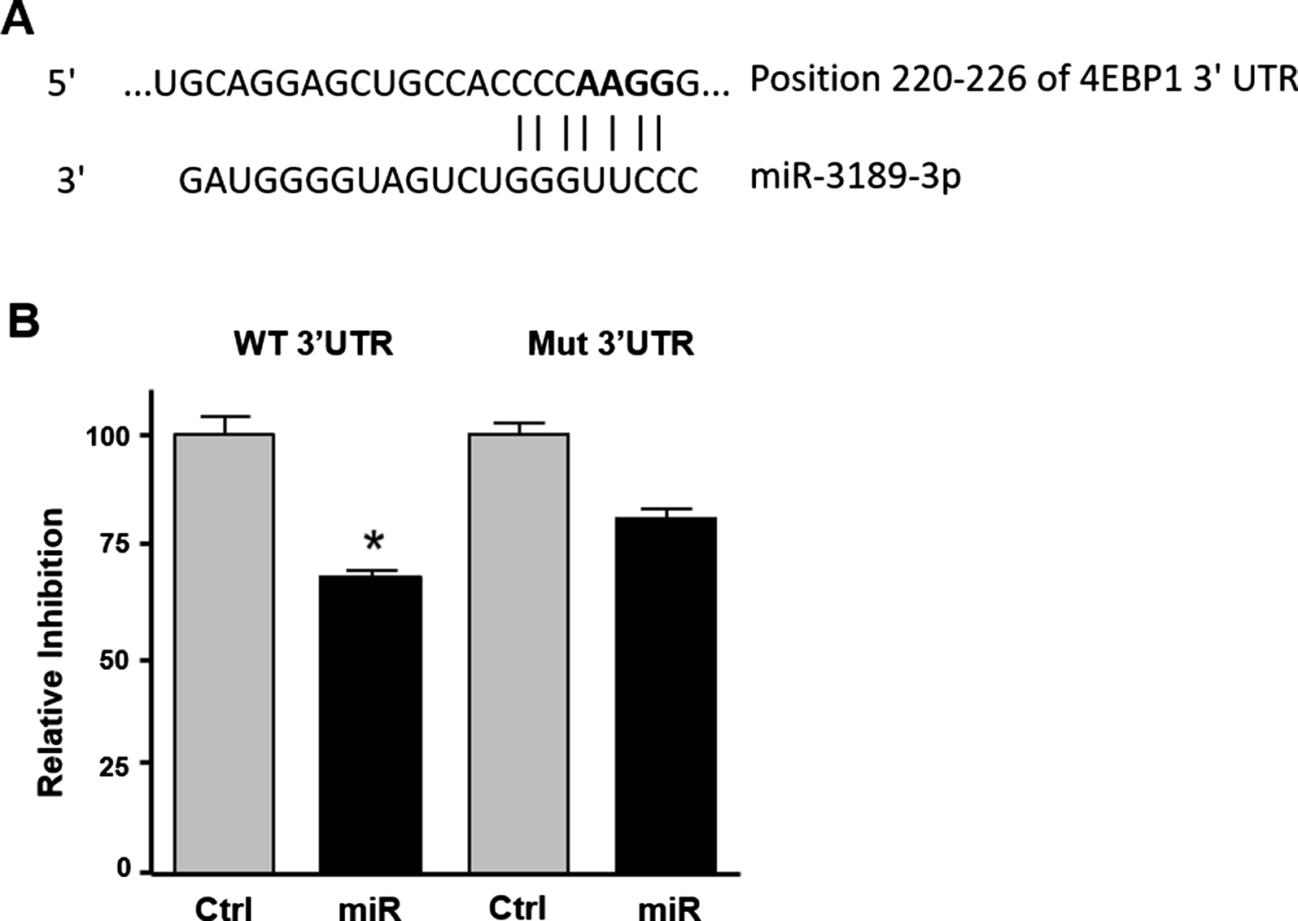
MiR-3189-3p directly targets 4EBP1 3′UTR. **A** Alignment of 4EBP1 3′UTR sequence with miR-3189-3p. In bold are indicated the mutated bases. **B** Luciferase assay of MDA-MB-231 cells co-transfected with miR-3189-3p (miR) or control (Ctrl) and either 4EBP1 3′UTR (WT 3′UTR) or the mutated 4EBP1 3′UTR (Mut 3′UTR). The scramble sequence of the miRNA was used as control. * Indicates statistical significance

Based on these results, a miRNA-target validation analysis was conducted using the 3′UTR of 4EBP1. The 3′UTR of 4EBP1 contains one putative binding site for the miR-3189-3p (Fig. 6A) and the direct binding of the miRNA was tested by a luciferase-based reporter assay. A reduction of approximately 30% (*p* < 0.05) of luminescence was observed in MDA-MB-231 cells transfected with miR-3189-3p mimics (Fig. 6B). Conversely, site-directed mutagenesis of the miRNA binding sequence in the 3′UTR showed an approximate 13% reversal of this reduction in luminescence signal, thereby demonstrating a sequence-specific direct binding of miR-3189-3p to 4EBP1 mRNA (Fig. 6B). The modest reversal effect of the mutated 3′UTR may indicate a general, binding-independent effect of the miRNA on translation, a phenomenon we observe when the expression plasmid is co-transfected with the miRNA (see below).

### Role of 4EBP1 in miR-3189-3p-mediated c-MYC downregulation

**Fig. 7:**
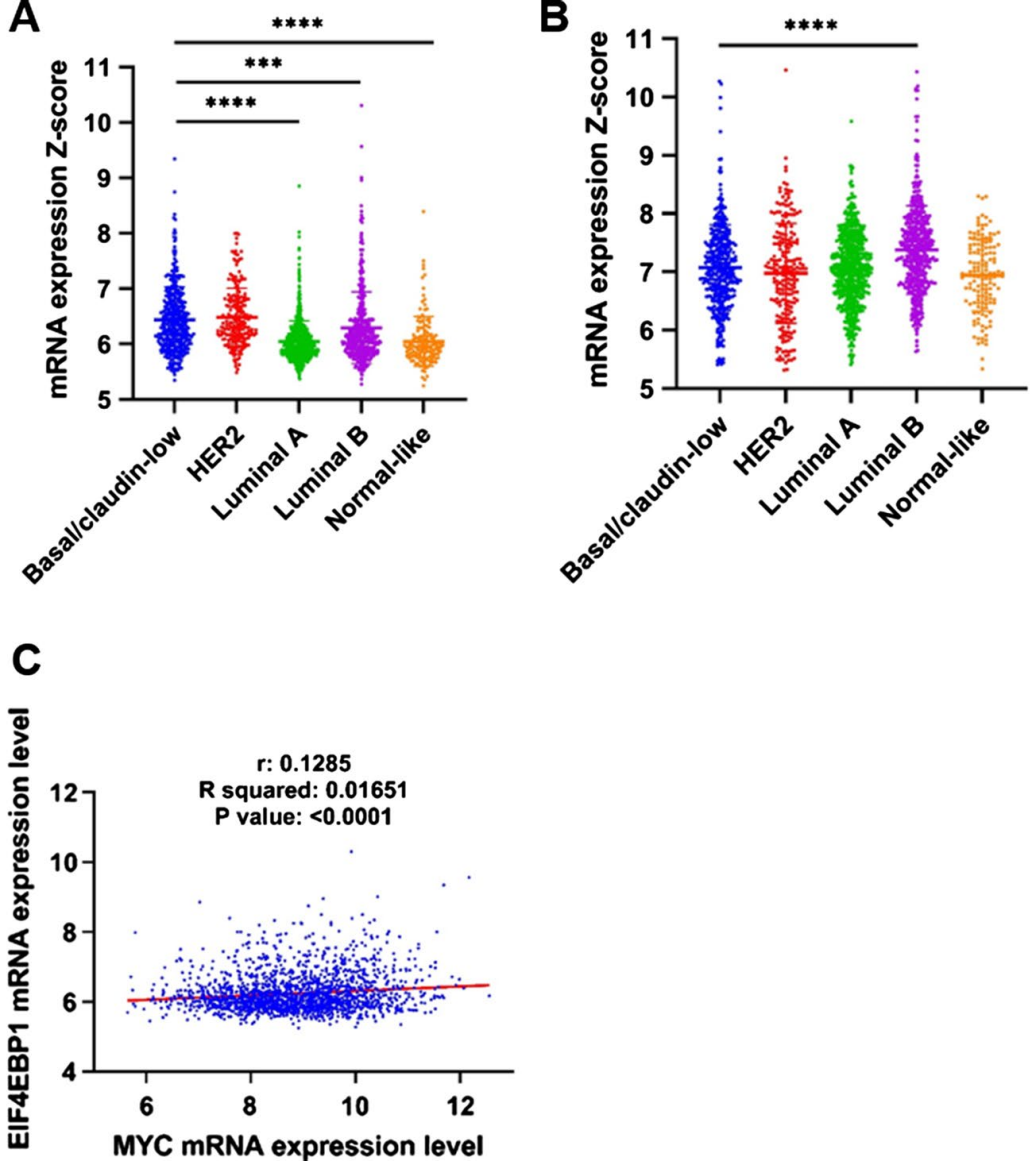
METABRIC data set analysis shows increased expression of 4EBP1, but not eIF4E, in TNBC compared to normal-like controls. Expression of 4EBP1 (**A**) and eIF4E (**B**) mRNAs in different subtypes of breast cancer (METABRIC). * indicates statistical significance. **C** Correlation between 4EBP1 and c-MYC mRNA expression in breast cancer tumors (METABRIC). * Indicates statistical significance. The association was measured using the Pearson correlation coefficient (R) and the P-value

**Fig. 8:**
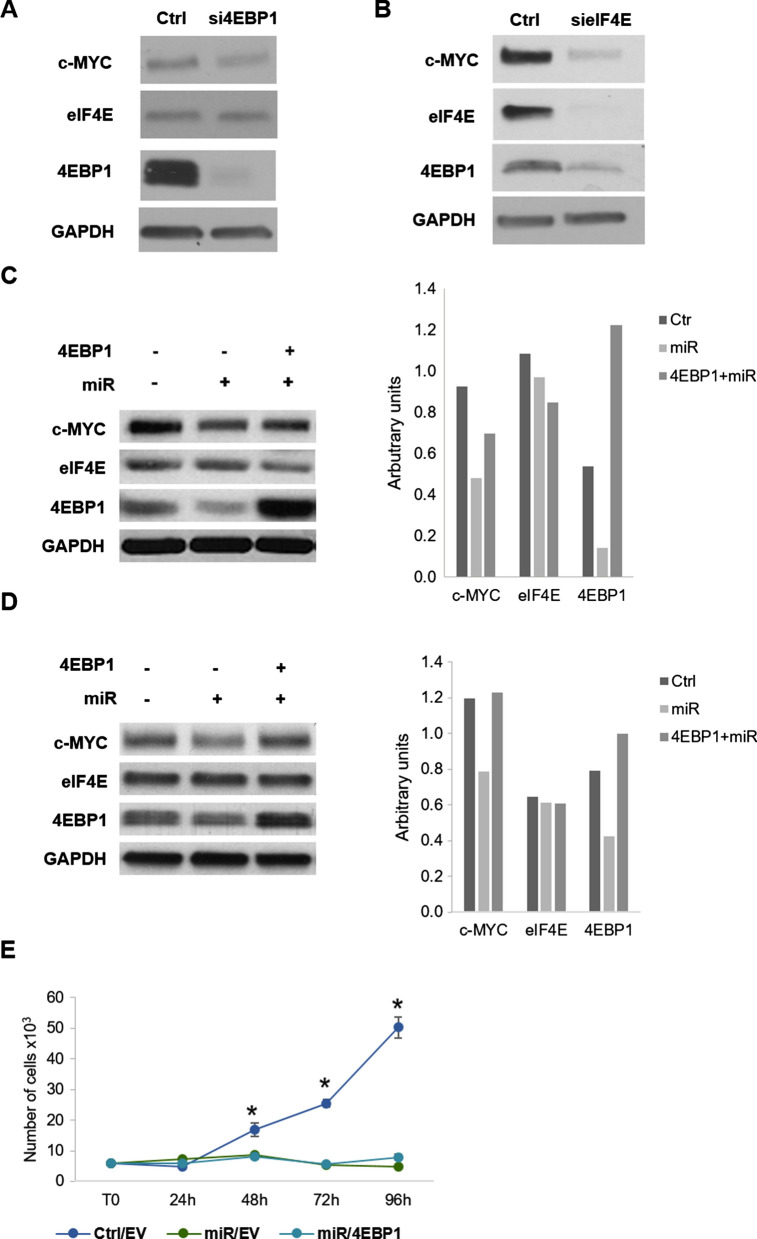
Role of 4EBP1 in the miR-3189-3p-mediated c-MYC downregulation. Western blots showing levels of the indicated proteins after silencing of 4EBP1 (**A**) or eIF4E (**B**). Cells transfected with the scramble RNA were used as control (Ctrl). Western blot showing c-MYC, eIF4E and 4EBP1 proteins in MDA-MB-231 cells transfected with miR-3189-3p or scramble control in the presence of serum (**C**) or in serum-free conditions (**D**). Where indicated, cells were co-transfected with 4EBP1 expression vector. GAPDH was used as a loading control for every blot. **E** Scatter plot showing proliferation of MDA-MB-231 cells transfected with miR-3189-3p (miR) and either pcDNA3 EV (EV) or 4EBP1. Cells transfected with scramble RNA and EV served as control. * Indicates statistical significance between Ctrl/EV and either miR/EV or miR/4EBP1

While 4EBP1 is generally thought of as an inhibitor of protein translation, it is often overexpressed in cancer, suggesting its cooperation with c-MYC in the tumorigenesis [6, 7, 23]. Using the genomic expression data from the METABRIC dataset, we found that 4EBP1 has the highest expression level in TNBC compared to other subtypes (Fig. 7A). Conversely, with the exception of the luminal B subtype, eIF4E expression did not show significant differences in TNBC compared to other breast cancer subtypes or normal-like tissue (Fig. 7B). Interestingly, there is a positive correlation between c-MYC and 4EBP1 gene expression level with an R squared of 0.0165 and a P value < 0.0001 (Figs. 5, 7C), indicating a possible co-regulation of these factors in TNBC. Therefore, we sought to better understand the role of 4EBP1 on miR-3189-3p-mediated downregulation of c-MYC expression in MDA-MB-231 cells. Using a siRNA-mediated approach, we first determined whether c-MYC levels are dependent on 4EBP1 expression. Indeed, results do show a decrease in c-MYC expression in 4EBP1-silenced cells compared to control siRNA transfected cells (Fig. 8A). Since the silencing of 4EBP1 decreases c-MYC protein levels, and since the inhibitory function of 4EBP1 on translation depends upon its binding to eIF4E [6, 7], we determined expression levels of eIF4E in our experimental conditions. We found that the protein levels of eIF4E were unchanged following 4EBP1 silencing, suggesting that the decrease in c-MYC expression was not due to the loss of eIF4E. Indeed, silencing eIF4E resulted in a dramatic reduction of both c-MYC and 4EBP1 proteins (Fig. 8B), indicating that the inhibitory effect of eIF4E on c-MYC could still be mediated by 4EBP1. Next, we reasoned that if downregulation of 4EBP1 following miRNA expression was critical for c-MYC translation, overexpression of 4EBP1 should restore c-MYC protein levels. Indeed, results in Fig. 8C indicate that overexpression of 4EBP1, via an expression plasmid, could, at least partially, prevent the miRNA-mediated downregulation of c-MYC.

So far, our data have shown that miR-3189-3p-induced downregulation of c-MYC translation involves the inhibition of 4EBP1 a key protein in the 5’CAP- mediated translation. Previous reports have found that under conditions of cellular stress, 4EBP1 can promote cap-independent, IRES-dependent protein translation [9, 24–26]. Furthermore, the c-MYC 5′UTR contains a functional IRES [27, 28]. Therefore, we were interested in testing whether miR-3189-3p could block the expression of c-MYC under conditions of cellular stress as other accessory proteins or mechanisms could be involved. As expected, also in serum-free condition, a significant decrease in the expression of c-MYC and 4EBP1 proteins was observed in cells treated with miR-3189-3p when compared to cells transfected with the control RNA (Fig. 8D) and the addition of 4EBP1 prevented this inhibition. However, transfection of 4EBP1 in the presence of miR-3189-3p did not prevent the miRNA-induced inhibition of proliferation, as shown in Fig. 8E.

### Negative effect of miR-3189-3p on cap-independent translation

**Fig. 9:**
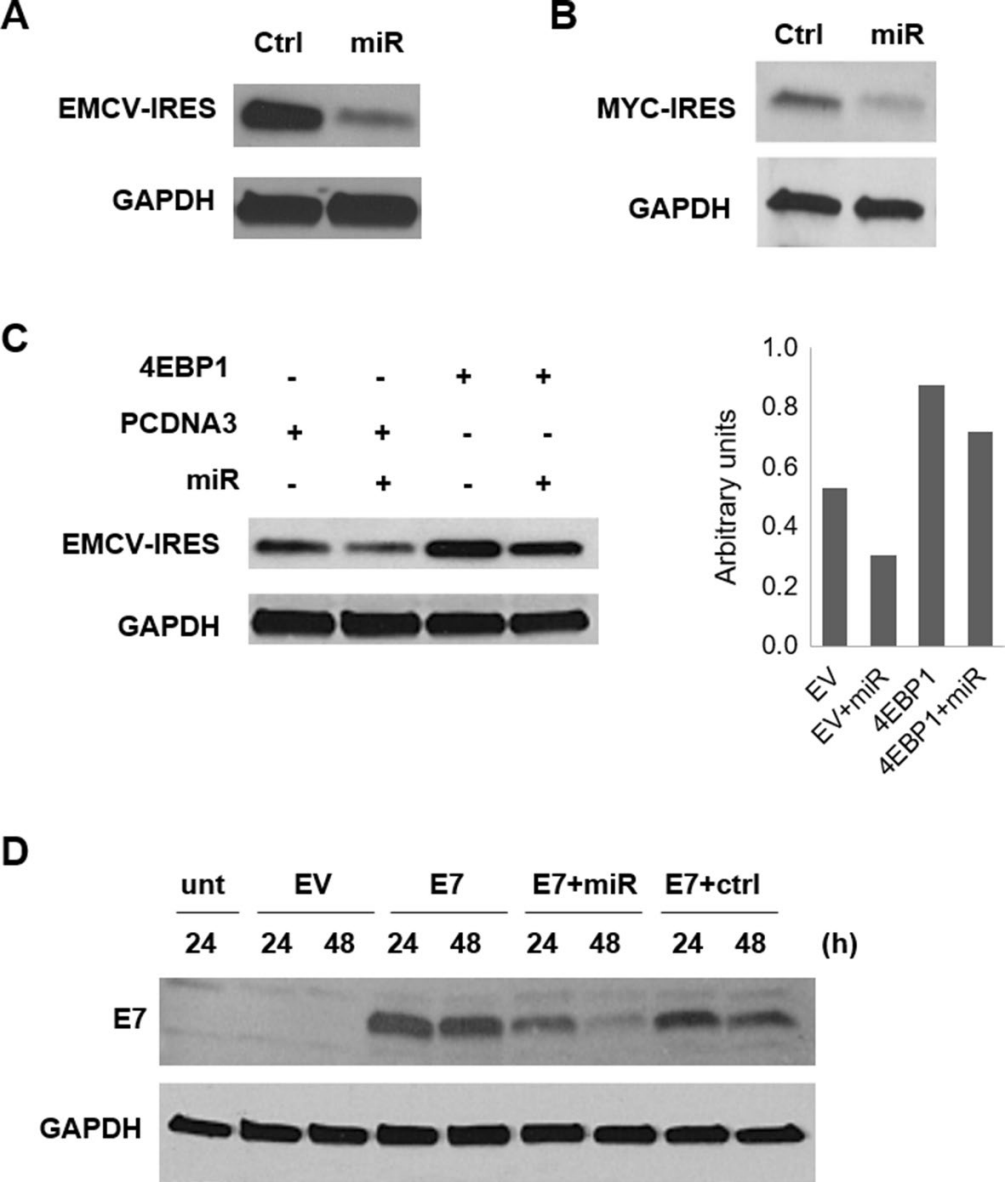
miR-3189-3p impairs IRES-dependent translation and m6A-dependent translation. Western blots to detect IRES-dependent translation of ECFP after co-transfection of MDA-MB-231 cells with pYIC/EMCV-IRES (**A**) or pYIC/c-MYC-IRES (**B**) and miR-3189-3p (miR) or scramble control (Ctrl).** C** Western blot to detect IRES-dependent translation of ECFP after co-transfection of MDA-MB-231 cells with pYIC/EMCV-IRES and miR-3189-3p or scramble control. Where indicated, cells were co-transfected also with 4EBP1 expression vector. EMCV- or MYC-IRES were detected using ECFP antibody. **D** Western blot of MDA-MB-231 cells co-transfected with miR-3189-3p (miR) or scramble control (Ctrl) together with a plasmid encoding a circular RNA containing the protein E7 under m6A translation (E7) or the empty vector (EV) for 24 and 48 h. GAPDH was used as a loading control for every blot

After establishing that miR-3189-3p impairs c-MYC protein expression in MDA-MB-231 under normal growth conditions and conditions of stress, we were interested in understanding whether the miRNA was interfering with cap-independent translation. As IRES sequences have been found in several mRNA, including c-MYC [24, 27], we tested if IRES-dependent translation of c-MYC was affected by miR-3189-3p. We utilized a bicistronic vector (pYIC) encoding a single transcript containing yellow fluorescent protein (EYFP, myc-tagged), viral IRES (EMCV-IRES), and blue fluorescent protein (ECFP, HA-tagged). In normal growth conditions, a cap-dependent translation will result in the expression of EYFP. However, in conditions that impair cap-dependent translation and favor IRES-dependent translation, the expression of ECFP should increase. MDA-MB-231 cells were co-transfected with the pYIC vector and either miR-3189-3p or the RNA scramble control. Results in Fig. 9A show much lower expression of EMCV-IRES-driven protein product in cells transfected with miR-3189-3p compared to control-transfected cells. We then replaced EMCV-IRES with the 395 bp c-MYC 5′UTR in the pYIC vector and confirmed the inhibitory effect of miR-3189-3p on this sequence as well (Fig. 9B). Next, we wanted to determine if 4EBP1 would increase IRES-dependent translation. Since EMCV-IRES has been found to be stronger than c-MYC IRES [29], we utilized the pYIC/EMCV vector to perform this experiment. We co-transfected cells with the miRNA or control and 4EBP1 expression plasmid or control plasmid (pcDNA3). As expected, miR-3189-3p downregulated the expression of EMCV-IRES driven protein and the addition of 4EBP1 at least partially rescued this downregulation (Fig. 9C).

Finally, we tested the effect of miR-3189-3p on another type of cap-independent translation, the m6A mRNA modification. To this end, we utilized a plasmid encoding a circular RNA with m6A driving the expression of the viral protein E7. Expression of E7 was evaluated in protein lysates obtained at 24 and 48 h after transfection with empty vector, E7 alone, E7 and miR-3189-3p, E7 and scramble control, or untransfected cells. Results clearly show an inhibitory effect of miR-3189-3p on E7 production, indicating an impairment in m6A-mediated translation in these cells (Fig. 9D).

### MiR-3189-3p sensitizes TNBC cells to doxorubicin

**Fig. 10:**
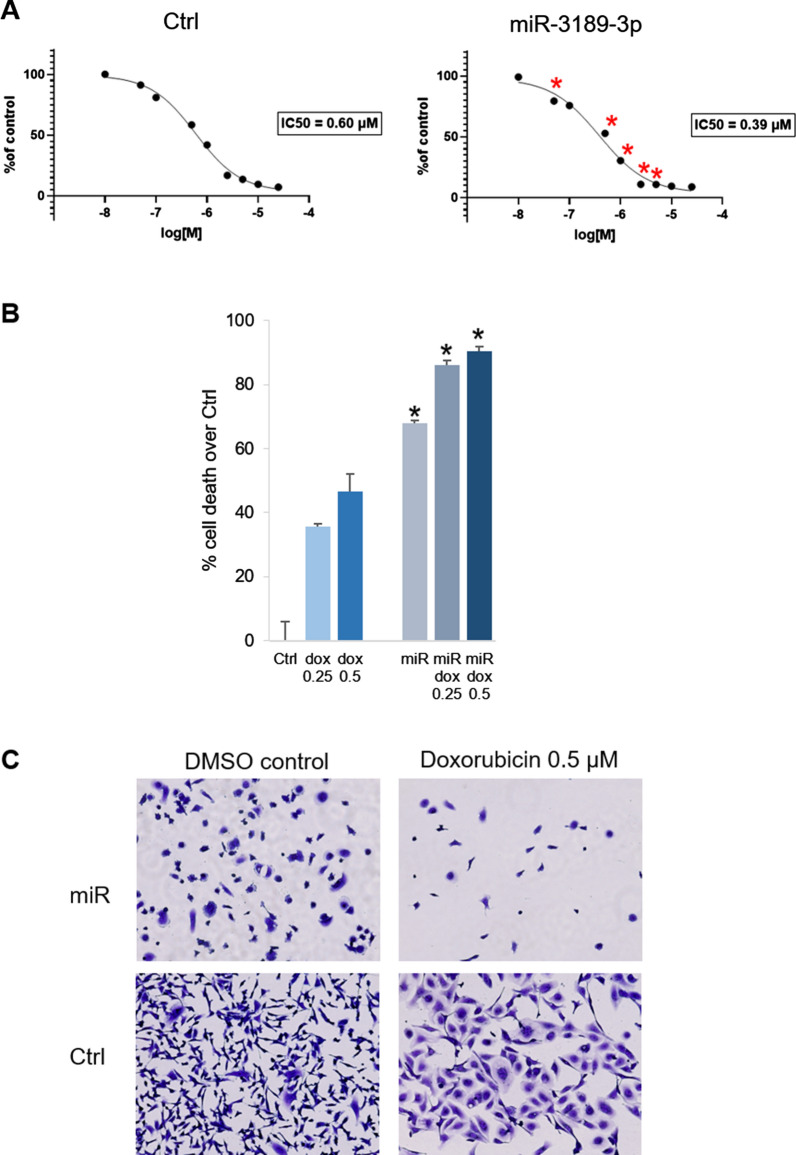
miR-3189-3p sensitizes MDA-MB-231 cells to doxorubicin. **A** Graphs indicating IC50 of doxorubicin in control-transfected (Ctrl) and miR-3189-3p-transfected (miR) cells. **B** Bar graph showing the percentage of cell death at the indicated concentrations of doxorubicin (dox) in control- or miR-transfected cells. **C** Representative images of Ctrl and miR-transfected cells after treatment with 0.5 µM doxorubicin or DMSO control. Asterisks in A and B indicate statistical significance between miR and control at the indicated concentrations of doxorubicin

In an effort to validate the potential therapeutic effect of miR-3189-3p, we determined its toxicity when used in combination with doxorubicin. Figure 10A shows the IC50 of doxorubicin in MDA-MB-231 cells transfected with scramble control (left panel) or with miR-3189-3p (right panel). Specifically, after 48 h of treatment with doxorubicin the IC50 was 0.6 µM and 0.39 µM for control-treated or miRNA-treated cells, respectively. The bar graph in Fig. 10B shows percentage of cell death after doxorubicin treatment at the concentrations of 0.25 and 0.5 µM. As previously observed, miR-3189-3p (miR) alone induces about 70% of cell death compared to the scramble control (Ctrl). Doxorubicin treatment killed about 50 and 44% more cells in miRNA-transfected compared to control cells. Representative images of cells stained with crystal violet after transfection with control or miR-3189-3p and treated with DMSO or doxorubicin at 0.5 µM are shown in Fig. 10C.

## Discussion

MiR-3189 is contained in the intron of Growth Differentiation Factor 15 (GDF15) [17, 30, 31] and its expression in various tissues is either low or undetectable [16, 17, 30–33]. Others and we have found a potent anti-cancer activity exerted by miR-3189-3p mimic on several types of tumors [17, 31, 34]. Recent literature suggests a potential role for miR-3189-3p in Parkinson’s disease [32], in osteoarthritis [33], and in endoplasmic reticulum stress response in various types of cancer cells [35]. Nevertheless, the function of miR-3189-3p in normal and pathologic conditions remains largely unknown. Similar to our previous data in glioblastoma [17], we confirmed the inhibitory role of miR-3189-3p in breast cancer cell proliferation and migration (Figs. 2 and 3A). Additionally, we demonstrated that miR-3189-3p impaired cell invasion in vitro (Fig. 3B), strengthening the anti-cancer function of this miRNA.

To better understand the mechanism behind the anti-proliferative effects of miR-3189-3p in TNBC, we investigated whether c-MYC could be regulated by this miRNA. C-MYC is an oncogenic protein overexpressed in many aggressive cancers, including breast cancer [13, 15]. In particular, our METABRIC analysis showed that c-MYC mRNA has the highest expression in TNBC compared to the other subtypes (Luminal A, Luminal B, HER2 + and Normal-like) (Fig. 4A). This transcription factor directs many aspects of cellular metabolism, including cell cycle progression, transformation, protein synthesis, and glucose uptake [36–39]. By upregulating c-MYC, cancers such as TNBC hijack these metabolic operations and promote unrestricted tumor growth and cell proliferation [36]. Interestingly, we consistently found lower levels of c-MYC protein expression in miR-3189-3p transfected MDA-MB-231 (Fig. 4B) and MDA-MB-468 (Fig. 5B) cells compared to controls, although c-MYC is not a direct target of the miRNA. Importantly, expression of miR-3189-3p in normal breast epithelial cells HME1 did not result in downregulation of c-MYC (Fig. 5C), suggesting that the activity of the miRNA on c-MYC is specific to tumor cells. Since c-MYC mRNA levels were unchanged upon expression of miR-3189-3p, and since the proteasome inhibitor MG132 failed to stabilize c-MYC protein (Fig. 4C), we excluded transcriptional inhibition and protein stability as miRNA-mediated mechanisms of c-MYC downregulation. A number of putative miR-3189-3p targets play a role in protein translation, including RNA-binding proteins and eukaryotic initiation factors. Following transfection of MDA-MB-231 cells with miR-3189-3p, we evaluated the expression of two putative targets of this miRNA, 4EBP1 and eIF4E. 4EBP1 is considered an inhibitor of cap-dependent translation and is one of the proteins responsible for blocking the inappropriate overexpression of proteins [6]. Active, dephosphorylated 4EBP1 inhibits the translation by binding to eIF4E and preventing the initiation of translation [40, 41]. For this inhibitory function, 4EBP1 is considered a tumor-suppressor; however, clinical tumor specimens repeatedly show an upregulation of 4EBP1 and c-MYC, suggesting a potential cooperation between the two proteins in cancer and tumorigenesis [6, 7, 23]. The contradictory role of elevated 4EBP1 in the presence of elevated c-MYC protein remains unclear. Our results showed downregulation of 4EBP1 by miR-3189-3p in TNBC cells (Fig. 5A and B) and a functional assay further demonstrated that the 4EBP1 mRNA is a direct target of miR-3189-3p (Fig. 6). Interestingly, although our data show that miR-3189-3p directly binds the 3′UTR of 4EBP1 mRNA, mutation of the miRNA binding site did not fully restore gene expression. This modest reversal effect may suggest a general, binding-independent effect of the miRNA on translation.

Since our METABRIC analysis showed that 4EBP1 mRNA has the highest expression in the TNBC subtype (Fig. 7A) and that a positive correlation between 4EBP1 and c-MYC mRNA exist in breast cancer (Fig. 7C), we were next interested in determining the role of 4EBP1 in the miR-3189-3p-mediated downregulation of c-MYC protein in MDA-MB-231 cells. The direct siRNA-mediated silencing of 4EBP1 revealed a dependence of c-MYC protein expression on 4EBP1 levels in these cells (Fig. 8A). This result is interesting, as downregulation of 4EBP1 should result in un-sequestered and fully active eIF4E capable of initiating efficient global translation, including translation of c-MYC. However, while c-MYC was downregulated, the levels of eIF4E remained unchanged, suggesting that the downregulation of c-MYC is independent from the expression of eIF4E. Alternatively, our data may indicate a dependence of eIF4E from 4EBP1 or, in other words, eIF4E requires a minimal expression of 4EBP1 to be functional [42, 43]. A direct correlation between eIF4E and 4EBP1 could indeed exist, since in cells where eIF4E is silenced, active and not-phosphorylated 4EBP1 is ubiquitinated and degraded by the proteasome, allowing the residual eIF4E to start the translation [44]. We did not evaluate general translation after eIF4E downregulation, nor the proteasomal degradation of 4EBP1, but we observed a simultaneous downregulation of 4EBP1 and c-MYC (Fig. 8B). This may indicate that in our experimental setting the residual eIF4E, if any, is not promoting c-MYC translation. We next reasoned that if c-MYC is dependent on 4EBP1 for translation, adding this protein back to miR-3189-3p-treated cells would restore c-MYC expression. Indeed, overexpression of 4EBP1 via an expression vector partially rescued c-MYC expression (Fig. 8C). Under conditions of cell stress, 4EBP1 is activated through dephosphorylation resulting in increased binding of eIF4E and consequent impairment of cap-dependent translation [45, 46]. However, under these conditions, a number of select mRNAs including MYC, could continue to be actively translated through cap-independent, IRES sequences [27, 47]. Since c-MYC 5′ mRNA contains an IRES, we tested whether our miRNA is affecting c-MYC expression in stress conditions, such as nutrient deprivation. Our results indicate that miR-3189-3p negatively affect c-MYC expression in the absence of serum (Fig. 8D) and that reintroducing 4EBP1 in the presence of the miRNA restored c-MYC expression (Fig. 8D), suggesting that 4EBP1 is one of the miR-3189-3p targets largely responsible for supporting c-MYC expression under stress. Nevertheless, re-expression of 4EBP1 in miRNA-transfected cells did not prevent the miRNA-mediated inhibition of proliferation (Fig. 8E).

The finding that miR-3189-3p reduces levels of c-MYC protein under stress is significant since upregulation of this oncogene is an important step in the survival response of tumor cells under nutrient deprivation or other types of stresses in the tumor microenvironment [6, 24, 26].

To gain insights into the role of miR3189-3p in cap-independent translation, we evaluated its effects on two types of translation, the IRES-dependent translation and the m6A-dependent translation, using specific plasmid constructs. To test IRES-mediated translation, we utilized a reporter plasmid in which either EMCV-IRES or c-MYC-IRES drives the expression of a fluorescent protein (ECFP) and found that miR-3189-3p efficiently downregulates expression of this protein compared to the miRNA negative control (Fig. 9A and B). Interestingly, the addition of 4EBP1 resulted in increased IRES-mediated translation (Fig. 9C), suggesting that 4EBP1 may support c-MYC expression under stress through the IRES-dependent translation.

N^6^-methyladenine (m6A) is a dynamic and reversible RNA modification abundantly present in mammalian mRNAs [48], including c-MYC mRNA [49, 50]. Since increased levels of m6A in c-MYC RNA can increase c-MYC expression [51, 52], we reasoned that our miRNA could impair also m6A-dependent translation. We transfected MDA-MB-231 cells with a plasmid encoding a circular RNA in which m6A methylations drive the expression of the EBV viral protein E7. Our data in Fig. 9D clearly show a reduction in E7 expression upon co-expression of the plasmid with miR-3189-3p.

We further thought to investigate a potential use of miR-3189-3p as a therapeutic approach in combination with the chemotherapeutic agent doxorubicin, one of the most common drug used to treat breast cancer. Therefore, we tested the toxicity of doxorubicin in cells transfected with the miRNA mimic or controls and found that the miRNA increased TNBC sensitivity to doxorubicin of about 35% (Fig. 10).

## Conclusions

In summary, our data strongly indicate an inhibitory effect of miR-3189-3p on the cap-dependent and cap–independent translation that result in decreased expression of c-MYC, both in normal growth conditions and in stress conditions. Considering that c-MYC is a potent survival factor in cancer cells, our findings may provide new therapeutic approaches that target the tumor microenvironment, often characterized by hypoxia and nutrient deprivation.

## Data Availability

The datasets analyzed during the current study were downloaded from the Molecular Taxonomy of Breast Cancer International Consortium (METABRIC) study available on the TCGA data portal (http://www.cbioportal.org/).
